# Effective Treatment of Rosacea and Telangiectasias Using IPL

**DOI:** 10.1111/jocd.70357

**Published:** 2025-07-30

**Authors:** Shaked Menashe, Irene Graciela Bermejo, Melina Lois, Leisa María Molinari, María Inés Reig

**Affiliations:** ^1^ The Department of Adult and Pediatric Plastic, Aesthetic and Reconstructive Surgery Shamir Medical Center Be'er Ya'akov, Shamir Medical Center Assaf Harofeh Tel Aviv Israel; ^2^ Advanced Dermatology Clinic Buenos Aires Argentina; ^3^ Advanced Skin Center Buenos Aires Argentina; ^4^ Centro Médico de la Piel Buenos Aires Argentina; ^5^ Avec Belle Clinic Río Grande Argentina

**Keywords:** IPL, rosacea, telangiectasias, VL/PL

## Abstract

**Background:**

To date, no definitive treatment exists for rosacea. Phototherapies, including intense pulsed light (IPL), have been reported to reduce its characteristic features of erythema and telangiectasias.

**Methods:**

This multicenter, retrospective study reviewed the charts of 82 patients with vascular and pigmented rosacea who underwent treatment with the Harmony XL Pro VL/PL Cooled Applicator. Lesion coverage was assessed from photographs taken before and 3–4 months after the last treatment session. Physicians assessed aesthetic improvement using the 5‐point Global Aesthetic Improvement Scale. Patients rated pain experienced during the session and satisfaction with outcomes. Treatment safety was monitored throughout.

**Results:**

A total of 82 patients with rosacea underwent up to four IPL treatment sessions. Mean patient age was 41.9 ± 15.2 years, and most were female (84.1%), with skin type II or III (96.1%) and with facial rosacea (93.9%). Clearance of > 75% was achieved in 69.5% of the patients, and the remaining 30.5% achieved 51%–75% clearance. Physician‐rated aesthetic improvement was optimal (64.6%) or good (34.1%); minimal change was reported for one lesion. Skin type III was associated with 3.59 times higher odds of achieving high clearance compared to skin type I or II (95% CI: 1.2–11.3). Patients were mostly very highly (95.1%) or highly satisfied (3.7%) with treatment outcomes. Most patients reported low (39.0%) to medium (46.3%) pain during treatment. Apart from a blister reported by one patient, no adverse events were reported.

**Conclusion:**

IPL is a safe, effective, and versatile light‐based modality for the treatment of vascular rosacea lesions in individuals of skin types I–III.

## 1 Introduction

Rosacea is a common chronic dermatosis, characterized by persistent centrofacial erythema, flushing, pustules, papules, and telangiectasias [1]. Ocular involvement, often preceding cutaneous manifestations, is common and can progress to severe corneal inflammation and scarring and even to visual loss [2]. The etiology of rosacea is likely multifactorial and has been linked to neurovascular dysregulation and aberrant immune responses, which lead to disruption of the epidermal barrier, vasodilation, and vessel leakage, as well as angiogenesis. These pathophysiological events translate to erythrocyte extravasation and chronic extravascular fluid accumulation, reduced vessel and connective tissue integrity, phenotypically presenting as chronic erythema, edema, vessel dilation, and telangiectasias, respectively [3]. Rosacea is most commonly reported in patients with fair‐colored skin but is likely underreported in populations with skin of color due to the indistinguishable erythema [4]. It can affect individuals of all ages, genders, and ethnicities, but is generally associated with morbidity in individuals in their fourth and fifth decades of life and is more common in females and in patients with northern or western European origins [4]. To date, there is no definitive cure for rosacea, but its effects can be minimized. Treatment is largely empirical and symptom‐focused and heavily reliant on topical or oral antibiotics, anti‐inflammatories, adrenergic agonists [5, 6].

Several phototherapies have proven successful in reducing erythema and eliminating telangiectasias and have been included in recent rosacea treatment guidelines and expert consensuses [7, 8]. For example, laser technologies using a wavelength selectively absorbed by the target chromophore and with a low absorption profile in the adjacent regions have been in extensive use for a wide range of pigmented skin lesions. Yet, this monochromatic coherent light modality carries the risk of post‐inflammatory hyperpigmentation, and its long‐term durability is often suboptimal. Intense pulsed light (IPL) is a flash lamp emitting noncoherent, noncollimated, broadband pulsed light in the range of 400–1200 nm. Integration of narrow‐band cutoff filters and careful setting of the pulse duration and fluence allow for treatment optimization to target specific chromophores in lesion types and sizes and at depths of interest while minimizing the risk of heat diffusion‐related thermal damage. For example, large and/or deeply seated vessels will require higher energy densities and longer pulses to achieve adequate heating and coagulation. IPL devices are indicated for a wide range of skin conditions and are considered particularly effective in treating pigmented and vascular lesions, attributed to the high absorption of light in this range by both hemoglobin and melanin [9, 10]. A retrospective assessment of the aesthetic improvements achieved with narrow‐band IPL (450–600 nm) in 100 patients presenting with vascular or pigmented lesions demonstrated the high efficacy of the regimen for both lesion types. At 3 months post‐treatment, the mean improvement score, measured using the 10‐point Global Aesthetic Improvement Scale (GAIS), was 8 for both subgroups and correlated with mean patient satisfaction scores (8.0 ± 0.8) [9]. Similar performance was achieved with a 550–650 nm IPL system used to reduce freckles, melasma, solar lentigo, post‐inflammatory hyperpigmentation, and post‐inflammatory erythema on the face, neck, and chest [10]. Within 2 months of treatment, notable aesthetic improvements in the pigmented skin lesions were documented (GAIS: 7.5 ± 1.2), which correlated with patient satisfaction ratings (7.3 ± 1.3). In both studies, patients reported no to low pain levels.

The current study conducted a comprehensive evaluation of the safety, performance, and patient tolerability of IPL technology as a therapeutic approach for managing facial rosacea symptoms in 82 patients.

## 2 Methods

This multicenter, retrospective analysis reviewed medical charts of patients with rosacea who underwent IPL treatment between 2022 and 2024, EC approval number 0203‐24‐ASF. Informed consent was obtained from all patients before study procedures were initiated.

### 2.1 Patients

Treatments were provided at one of four medical centers to adult female and male patients presenting with vascular and pigmented facial rosacea–associated lesions. Exclusion criteria included patients with Fitzpatrick phototype V–VI, patients unable to refrain from tanning or using a tanning bed within the preceding 30 days, hypopigmentation, any inflammatory skin condition or autoimmune skin disease in the target site, cancer and/or on cancer drug therapy, a history of keloid scarring, pregnant, uncontrolled diabetes, or epilepsy. In addition, patients treated within the past 3 months with St. John's wort, isotretinoin (Roaccutane) within the past 6 months or tretinoin (Retin A) in the past 2 weeks were also excluded.

### 2.2 Treatment Procedure

Lesions were treated with the VL/PL Cooled Applicator (Harmony XL Pro, Alma Lasers Ltd., Caesarea, Israel), operating within the 540–950 nm wavelength range. The incorporated proprietary advanced fluorescence filter (AFT) ensures delivery of uniform energy and controlled peak power throughout each pulse. Treatment parameters were 3 cm^2^ spot size, 10, 12, or 15 ms pulse duration, and 5–30 J/cm^2^ fluence. Treatment settings were adjusted to best suit patient skin type and lesion color, per recommendations in the device user manual (Table 1).

**TABLE 1:** VL/PL treatment parameters.

Skin type	Patients	Fluence range^a^ (J/cm^2^)	Pulse width range^b^ (ms)
I	3	16–18	10 or 12
II	27	15–18^c^	10 or 12
III	47	15–18	10, 12, or 15
IV	5	16–18	10 or 12

^a^ High fluence aligns with the vascular lesions protocol.

^b^ Short pulse width aligns with the vascular lesions protocol.

^c^ Exception: One case ONLY with a fluence of 20 J/cm^2^.

In cases of facial lesions, the emission was homogenously delivered to the entire face, while for body lesions, the surrounding area was masked. A thin layer of ultrasound gel was applied to target sites prior to treatment. Affected areas were subjected to up to four treatment sessions, conducted at 4‐week intervals.

Patients were instructed to avoid sun exposure and to apply sun protection factor ≥ 50 sunscreen on the treated area during the treatment and follow‐up periods. A follow‐up assessment was performed 3–4 months after the last treatment session.

### 2.3 Efficacy Evaluation

An independent, blinded expert dermatologist at each treatment center, rated vascular lesion coverage in photographs taken before and 3–4 months after the last treatment session, using a four‐point clearance scale, where 1 indicated 0%–25% clearance, 2 indicated 26%–50% clearance, 3 indicated 51%–75% clearance, and 4 indicated > 75% clearance as compared to baseline. Physicians used the GAIS to rate the aesthetic improvement, where “1” indicated “worse than before treatment” and “5” indicated “optimal improvement.” Patient satisfaction was scored using a 5‐point scales, with “1” indicating maximal dissatisfaction and “5” indicating maximal satisfaction.

### 2.4 Safety Evaluation

Adverse events were monitored and recorded throughout the treatment and follow‐up periods. In addition, at the end of each treatment session, patients were asked to rate the level of pain experienced during the session, using the 11‐point numeric pain rating scale (NPRS), with “0” indicating “no pain” and “10” indicating “severe pain.”

### 2.5 Statistical Analysis

Descriptive statistics were used to summarize study data, with continuous variables presented as mean and standard deviation, and categorical variables presented as count and percentage. Agreement between GAIS and clearance, representing the agreement between expert dermatologist and physician evaluations, was calculated using the Kappa Agreement test.

A logistic regression model was applied to identify factors associated with high clearance.

A two‐sided *p* value of < 0.05 was considered statistically significant for all analyses.

Analyses were performed using R version 4.4.1 (R Foundation for Statistical Computing, Vienna, Austria).

## 3 Results

A total of 82 patients presenting with vascular rosacea lesions underwent up to four IPL treatment sessions (mean: 2.4 ± 0.8). Mean patient age was 41.9 ± 15.2 years; 84.1% were female, and most were with skin type II (35.1%) or III (61.0%). The majority of patients presented with facial rosacea (93.9%). Other treated areas included the nasal region (2.4%), arm (1.2%), face and neck (1.2%), and neck only (1.2%). For most patients, this was their first experience with laser or light‐based therapy for rosacea (93.9%). Patient demographics and rosacea history by clearance level are presented in Table 2.

**TABLE 2:** Patient demographics and baseline characteristics.

Gender, *n* (%)	
Female	69 (84.1)
Male	13 (15.9)
Age (years)	—
Mean (CI)	41.87 (38.62, 45.12)
Median (Q1, Q3)	40.00 (32.00, 52.00)
Min–Max	19–75
Body area, *n* (%)	
Arm	1 (1.2)
Face	77 (93.9)
Face and neck	1 (1.2)
Neck	1 (1.2)
Nose	2 (2.4)
Skin type, *n* (%)	
I	3 (3.7)
II	27 (32.9)
III	47 (57.3)
IV	5 (6.1)
Prior laser treatment, *n* (%)	
No	77 (93.9)
Yes	5 (6.1)
Number of treatments, *n* (%)	
1	12 (14.6)
2	29 (35.4)
3	37 (45.1)
4	4 (4.9)

Clearance of > 75% was achieved in 69.5% of the patient cohort; the remaining 30.5% of the cohort showed 51%–75% clearance compared to baseline (Table 2). Physician‐rated aesthetic improvement (GAIS) as optimal (64.6%) or good (34.1%); minimal change was reported for one lesion only (1.2%) (Table 3).

**TABLE 3:** Treatment outcomes.

Outcome	Clearance (%)		*p*
	51%–75%	> 75%	
	(*N* = 25, 30.5%)	(*N* = 57, 69.5%)	
*Efficacy*			
GAIS, *n* (%)			0.0005
3	1 (4.0)	0 (0.0)	
4	24 (96.0)	4 (7.0)	
5	0 (0.0)	53 (93.0)	
Satisfaction, *n* (%)			0.1744
3	1 (4.0)	0 (0.0)	
4	0 (0.0)	3 (5.3)	
5	24 (96.0)	54 (94.7)	
*Safety*			
Pain level, *n* (%)			0.007
Low	15 (60.0)	17 (29.8)	
Medium	5 (20.0)	33 (57.9)	
High	5 (20.0)	7 (12.3)	
Adverse events, *n* (%)			1
Blister	0 (0.0)	1 (1.8)	
*Efficacy*			
None	25 (100.0)	56 (98.2)	

A high level of agreement between percent clearance and GAIS was demonstrated; nearly all subjects in the > 75% clearance group (93.3%) were rated as highly improved (GAIS = 5), and 100% of the 51%–75% clearance group had GAIS scores of 3 or 4 (Kappa = 0.88 95% CI (0.77–0.99)) (Table 4).

**TABLE 4:** Correlation among efficacy measures.

Assessment scale	Percent clearance		*p*
	51%–75%	> 75%	
	(*N* = 25)	(*N* = 57)	
GAIS, *n* (%)			< 1e−04
3 + 4	25 (100.0)	4 (7.0)	
5	0 (0.0)	53 (93.0)	
Satisfaction, *n* (%)			1
3 + 4	1 (4.0%)	3 (5.3)	
5	24 (96.0%)	54 (94.7)	

Among patients with lower clearance, 62.5% had undergone two treatment sessions, whereas the majority (58.5%) of those with high clearance had undergone 3–4 treatments (*p* = 0.004).

Among patients with lower clearance, 60.0% reported low pain levels, whereas the majority of those with high clearance (57.9%) reported experiencing medium pain levels (*p* = 0.0035).

Most patients reported on low (39.0%) to medium (46.3%) pain levels during treatment sessions. Apart from a blister reported by one patient, no other adverse events were reported.

**FIGURE 1:**
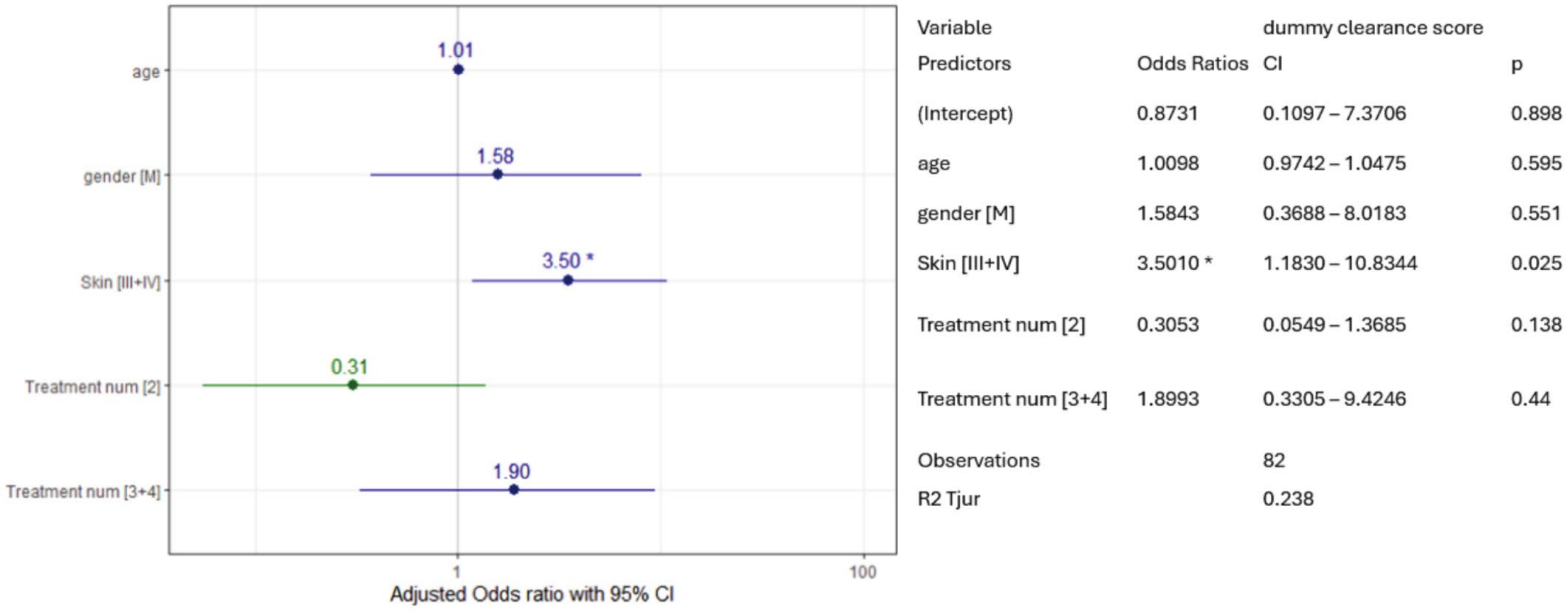
Logistic regression for clearance > 75% demonstrating “Skin Type” as the only predictor for clearance. Data analysis conducted to find the relationships between “clearance” and the various factors. As seen, the only statistically significant relationship was found for “Skin Type”.

A logistic regression analysis conducted to identify factors associated with > 75% clearance considered age, gender, skin type (I + II vs. III + IV), and the number of treatments as potential factors. Among these variables, the only significant predictor was skin type, where individuals with skin type III had 3.59 times higher odds of achieving high clearance compared to those with skin type I or II (OR: 3.59, 95% CI: 1.2–11.3) (Figure 1).

**FIGURE 2:**
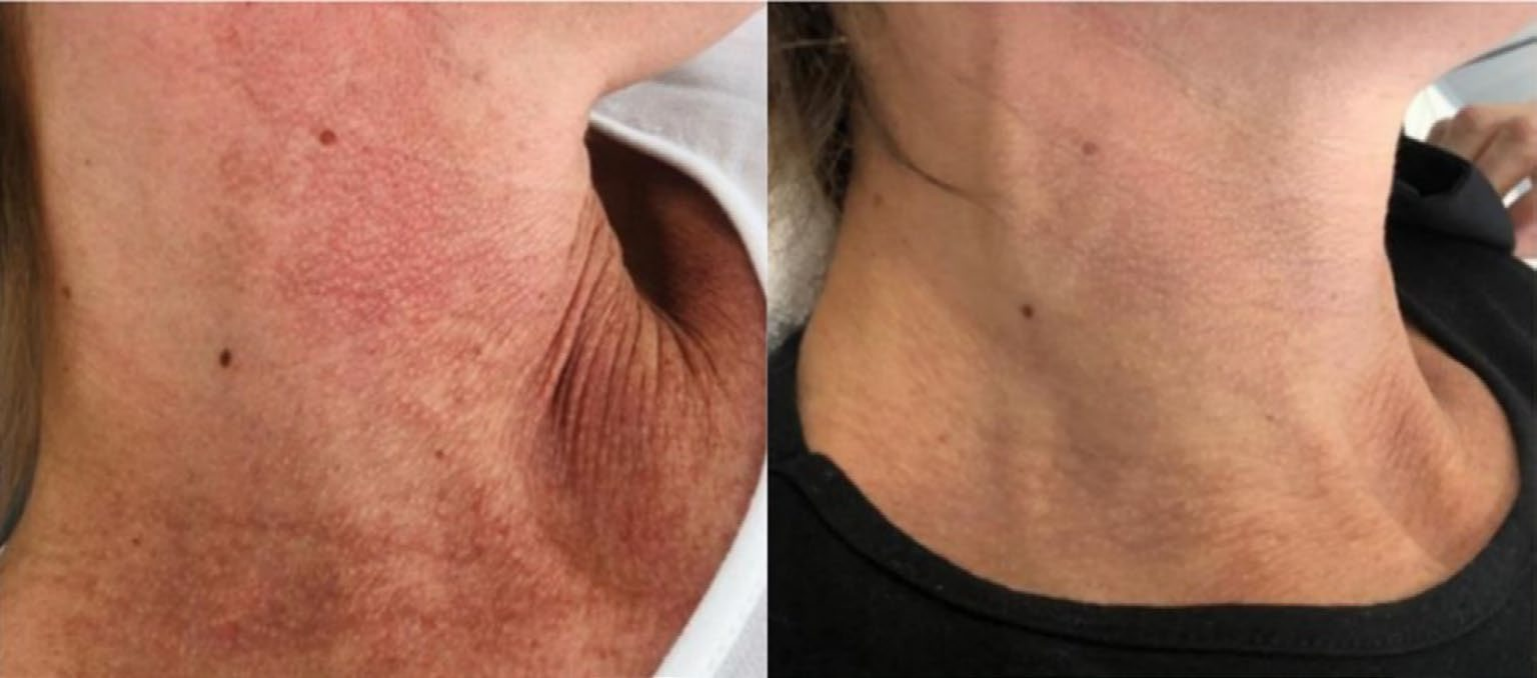
Clearance of neck rosacea. Before (left) and 3 months following (right) four intense pulsed light treatment sessions demonstrating > 75% clearance.

**FIGURE 3:**
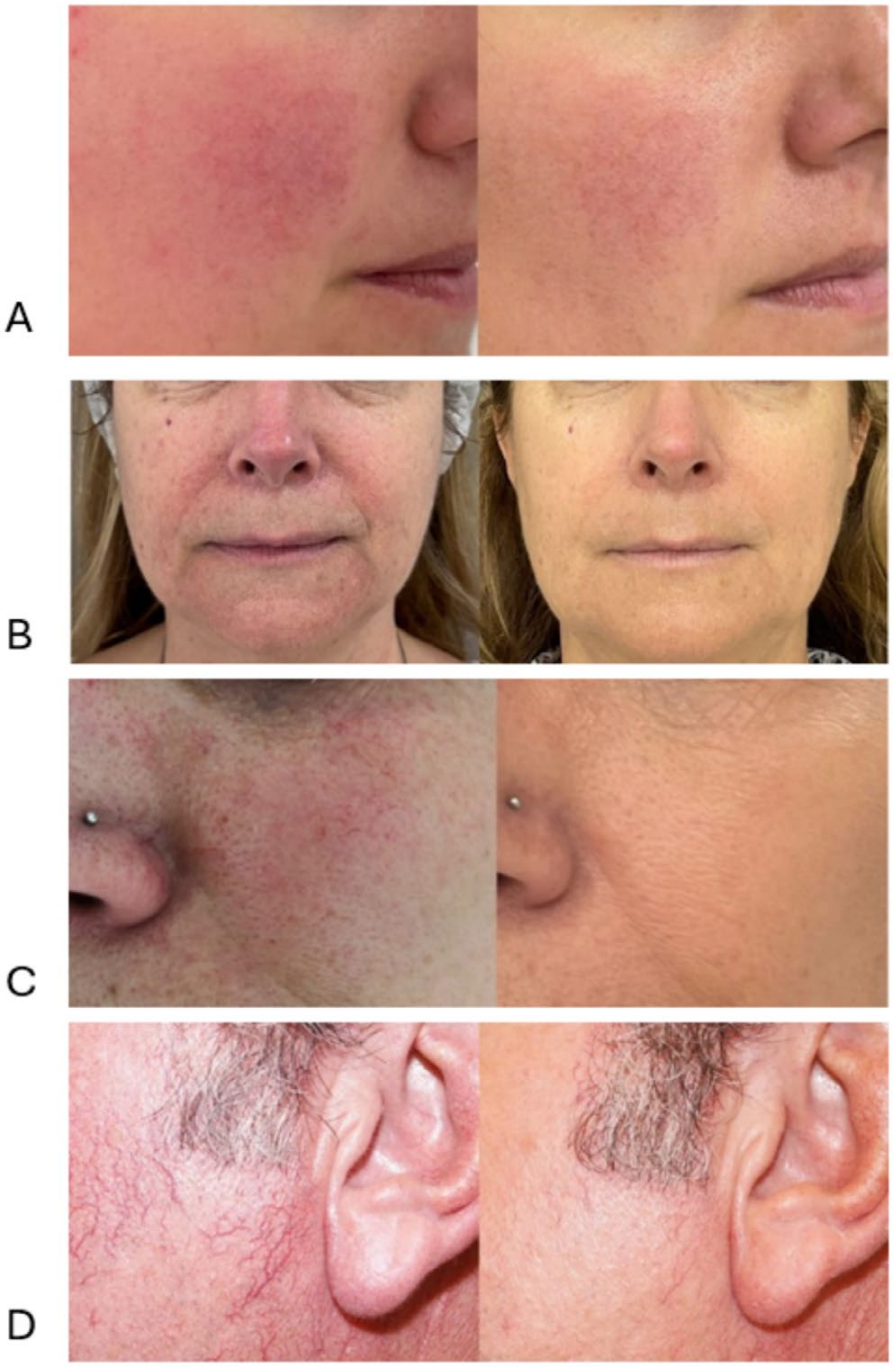
Clearance of face rosacea. Images taken before (left) and after (right) intense pulsed light (IPL) treatment of facial rosacea. (A) Assessment of cheek rosacea 3 months after four IPL sessions showed 51%–75% clearance of vascular lesions. (B) Assessment of perioral and chin rosacea 3 months after three IPL sessions showed > 75% clearance of vascular lesions. (C) Assessment of cheek telangiectasias and rosacea 4 months after four IPL sessions showed > 75% clearance of lesions. (D) Assessment of temple telangiectasias 4 months after a single IPL session showed > 75% clearance.

**FIGURE 4:**
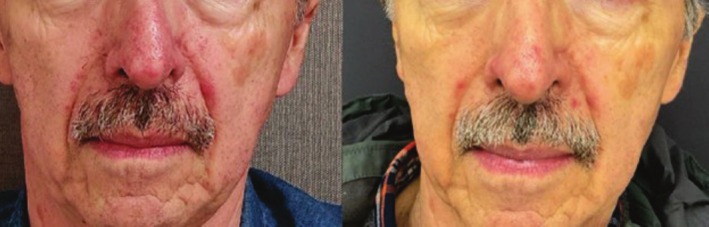
Clearance of nasal and perioral rosacea. Before (left) and 4 months following (right) three intense pulsed light treatment sessions demonstrating > 75% clearance of nasal and perioral rosacea and vascular lesions.

Examples of visible clearance of rosacea lesions on the neck, face, and nose are presented in Figures 2, 3, 4. Subject satisfaction was high for both levels of clearance, with 95.1% very highly satisfied (score 5/5), 3.7% highly satisfied (score 4/5), and 1.2% moderate satisfaction score (3/5).

## 4 Discussion

This retrospective chart review demonstrated the high safety and efficacy profile of IPL for treatment of vascularized rosacea lesions. Patients reported low to moderate pain levels and only one suffered a mild side effect that resolved within up to 4 weeks. Outcomes were favorable, with 70% of the patient cohort achieving 76%–100% clearance in the treated area, while the remaining patients achieved at least 50% clearance. Patients with skin type III showed higher responsiveness to treatment than patients with skin type I or II. The clearance outcomes were reinforced by GAIS ratings, indicating that the two forms of assessments provide a consistent picture of treatment efficacy. Importantly, patient satisfaction was very high in both subcohorts, demonstrating higher physician vs. patient expectations from IPL treatment. The clearance results aligned with previous reports of IPL performance and safety in the vascular and pigmented lesion arena. For example, in a 188‐patient cohort presenting with facial vascular lesions treated, 75%–100% clearance was achieved by 174 patients following up to four sessions of 550–590 nm IPL [11]. Treatment was well tolerated, and no scarring or permanent side effects were reported. In a 102‐patient cohort with mild to severe rosacea, 530 nm IPL (10–30 J/cm^2^) was delivered at 1‐ to 3‐week intervals to treat flushing and redness, while a 420‐nm filter (10–20 J/cm^2^) was used for patients also suffering from acneiform breakouts and telangiectasias. A retrospective analysis of treatment outcomes found that after a mean of 7.2 sessions, delivered at 1–3‐week intervals, 80% of the patients showed reduced erythema, 78% reported improved flushing, and 72% reported fewer acneiform breakouts [12]. A 51% reduction in telangiectasias was also noted. No complications or adverse reactions were reported. Schroeter et al. reported an average telangiectasia clearance rate of 77.8% in the 508 sites they monitored following an average of 4.1 sessions per patient of 515–1200 nm IPL [13]; results were sustained throughout the 51.6‐month follow‐up period. Spectrophotometric evaluation of the skin color of 12 patients with rosacea treated with IPL 550–670 nm measured significant improvements in the CIELAB color space in both erythematotelangiectatic and papulopustular rosacea patients [14].

Laser and light‐based therapies are being exploited for a growing number of clinical indications, particularly in the field of dermatology. The common thread running through these modalities is their ability to target specific chromophores, whose activation leads to selective photothermolysis of tissues of interest. In the case of rosacea, light therapy targets the dilated superficial blood vessels underlying the hallmark chronic erythema and telangiectasias. The 540–950 nm rays delivered in the current study are absorbed by circulating deoxyhemoglobin and metahemoglobin, which have peak absorbance at 542 and 577 nm, respectively. The generated heat energy induces localized vascular destruction. The near‐infrared component of the emitted spectrum is effectively absorbed by water within targeted tissues, resulting in mild coagulation and subsequent inflammatory responses. These, in turn, activate resident fibroblasts, which produce collagen, suggested to improve skin texture [15]. Use of this technique addresses the fine balance required between treatment safety and efficacy by the in‐motion safe delivery of the IPL, which is extremely important for darker skin types (low fluence and high repetition rate), thereby minimizing hyperpigmentation reactions and therefore extends candidacy criteria to include patients with skin of color.

Apart from the selection of the most appropriate wavelength, careful setting of the pulse duration (10–15 ms) to be shorter than the thermal relaxation time (TRT) of the target chromophore (A 0.3‐mm blood vessel has a 40‐ms TRT, and increasing the diameter to 1 mm brings its TRT up to 500 ms [16]), provides for more uniform energy distribution and minimizes the risk of heat diffusion‐related thermal damage. The undesirable effects of IPL pulses exceeding the TRT of off‐target chromophores can be offset by low fluences, which deliver less energy per unit area. In addition to the above, the AFT technology integrated in the Harmony XL PRO system converts otherwise wasted UV rays into the desired spectrum, which maximizes energy delivered per pulse, thereby minimizing the risk of adverse events which can occur on exposure to nontherapeutic energy densities and uncontrolled peaks. This, together with the integral cooling tip designed to maximize light penetration and minimize superficial burns and patient discomfort, eliminates risks associated with UV exposure and ensures tolerability. These features are of particular importance, considering the recent meta‐analysis comparing several laser and light‐based therapies, which found IPL to be as effective as other laser and light‐based therapies, with corresponding patient satisfaction levels, but also the most painful modality [17]. In the current cohort, the vast majority of patients reported low to moderate pain levels. Finally, IPL technology is highly versatile, with multiple features that can be tailored to skin and lesion types to enhance treatment selectivity while minimizing adverse reactions.

Limitations of this work included its retrospective design and lack of a control group. However, the consistent outcomes achieved by all four operators, suggest treatment robustness. Nonetheless, well‐controlled and long‐term studies on the safety, tolerability, and efficacy of light‐based therapies for rosacea are still in order. In addition, the improved outcomes in patients with skin type III vs. II may have been a corollary of the subjective assessment tools used to measure treatment outcomes; as vascularity and pigment are more prominent in fair‐skinned individuals, the improvement may have been less conspicuous to the naked eye. Follow‐up studies should consider use of objective colorimetric tools to determine treatment outcomes. Further, while the Harmony XL Pro device cutoff filter avoided the UV to green light range, rendering it suitable for dark‐skinned individuals, the majority of patients in this cohort were with Fitzpatrick skin type II or III, raising questions regarding the generalizability of the observed results to additional phototypes. Of note, four of the five patients with Fitzpatrick skin type IV in the current cohort achieved optimal results; one last patient achieved 51%–75% clearance. All five were highly satisfied with the aesthetic improvement. Three of the five patients scored their pain during treatment ≥ 6, a report which should be factored in when selecting treatment settings for patients with dark‐toned skin.

In conclusion, IPL proved to be a safe, effective, and versatile light‐based modality for treatment of vascular rosacea lesions. The integrated cutoff filter allowed for tight control over the lamp wavelength, which targeted the superficial vascular lesions while sparing adjacent structures.

At the same time, careful setting of fluence and pulse duration minimized the risk of heat diffusion‐related thermal damage and pain.

## Author Contributions

I.G.B., M.L., L.M.M., and M.I.R. performed the research. S.M. wrote the paper.

## Conflicts of Interest

The authors declare no conflicts of interest.

## Data Availability

Research data are not shared.
